# A Remarkable Clinical Response to Pembrolizumab in a Rare Spindle Cell Carcinoma of the Lung

**DOI:** 10.31662/jmaj.2019-0015

**Published:** 2019-12-27

**Authors:** Kyoji Tsurumi, Yosuke Kawashima, Junichi Akahira, Ryohei Saito, Yukihiro Toi, Atsushi Nakamura, Shinsuke Yamanda, Yuichiro Kimura, Yoshihiro Honda, Shunichi Sugawara

**Affiliations:** 1 Department of Pulmonary Medicine, Sendai Kousei Hospital, Sendai, Japan; 2 Department of Pathology, Sendai Kousei Hospital, Sendai, Japan

**Keywords:** Spindle cell carcinoma, Pembrolizumab, PD-L1, Sarcomatoid carcinoma

## Abstract

Spindle cell carcinoma of the lung consists of only spindle-shaped tumor cells, and accounts for approximately 13.3% of all sarcomatoid carcinomas (SCs), which are a rare subtype of poorly differentiated non-small cell lung cancer (NSCLC). Spindle cell carcinoma of the lung has very poor prognosis owing to resistance to chemotherapy and radiotherapy. This case report describes a 76-year-old man who presented with complaints of dry cough and right-sided neck pain and was later diagnosed with spindle cell carcinoma of the lung. He had a medical history of type 2 diabetes, angina pectoris, atrial fibrillation, hypertension, hyperlipidemia, and hepatitis B and a 20 pack-year history of smoking. A computed tomography (CT) scan revealed a mass with a thick-walled cavity in the right upper lobe of the lung. His neck pain was consistent with PET-CT images, indicating metastases due to invasion of lung cancer cells. The expression of PD-L1 in more than 90% of the tumor cells of the lung biopsy tissue led to the administration of pembrolizumab. The lung and metastatic tumors dramatically decreased in size after 9 weeks, and no tumor regrowth was observed over 11 courses of pembrolizumab administration. To the best of our knowledge, there are no previous reports describing the use of pembrolizumab for spindle cell carcinoma of the lung. This case report suggests that immunotherapy could be a promising treatment option for rare types of lung cancers, including spindle cell carcinoma.

## Introduction

Sarcomatoid carcinoma (SC) is a rare subtype of poorly differentiated non-small cell lung cancer (NSCLC), accounting for 0.1%–0.4% of all malignant tumors of the lung ^(1)^. According to the latest World Health Organization (WHO) classification, SC comprises of five histopathological subtypes, namely, pleomorphic carcinoma, spindle cell carcinoma, giant cell carcinoma, carcinosarcoma, and pulmonary blastoma. Spindle cell carcinoma consists of only spindle-shaped tumor cells and accounts for approximately 13.3% of all SCs ^(2)^. Spindle cell carcinoma of the lung often affects older male smokers, and the prognosis is very poor due to resistance to chemotherapy and radiotherapy ^(3)^. To the best of our knowledge, we are the first to report a case of spindle cell carcinoma of the lung treated with pembrolizumab.

## Case Report

**Figure 1.**
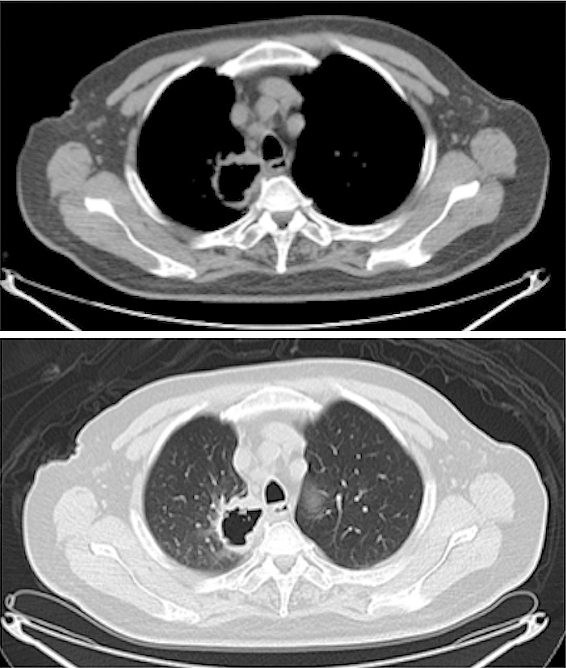
Computed tomography (CT) image reveals a mass with a thick-walled cavity in the right upper lobe of the lung.

A 76-year-old man visited the hospital complaining of dry cough and right-sided neck pain. He had a past medical history of type 2 diabetes, angina pectoris, atrial fibrillation, hypertension, hyperlipidemia, and hepatitis B and a 20 pack-year history of smoking. A computed tomography (CT) scan showed a mass with a thick-walled cavity in the right upper lobe of the lung (Figure 1). A transbronchial lung biopsy revealed proliferation of spindle cells which were immunohistochemically positive for cytokeratin and vimentin.

**Figure 2.**
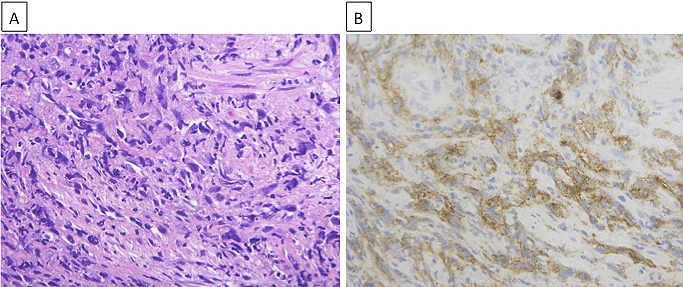
A. Hematoxylin and eosin (H&E) staining of the lung tumor tissue. B. Immunohistochemical expression of programmed death ligand-1 (PD-L1). PD-L1 was observed in more than 90% of tumor cells in the lung biopsy tissue.

**Figure 3.**
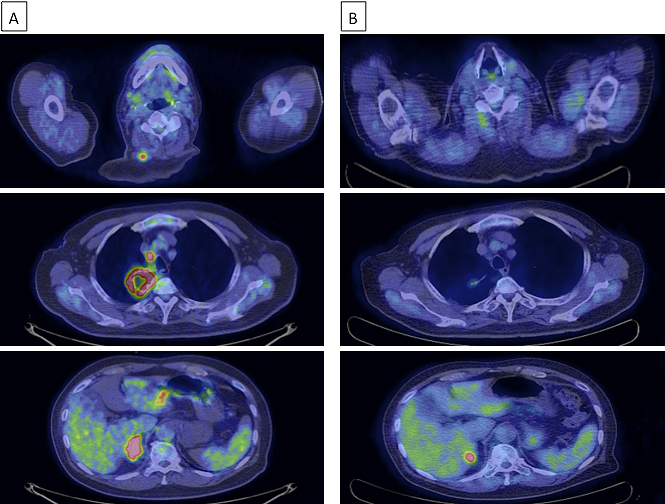
Images from positron emission tomography (PET)-CT scan of the patient. A. Fluorodeoxyglucose uptake in the mediastinal lymph node, right adrenal gland, and right trapezius muscle. B. A significant decrease in lung and metastatic tumor size as indicated by reduced fluorodeoxyglucose uptake, 21 weeks after treatment began.

Although the limited size of the biopsy specimen made a thorough analysis difficult, we confirmed spindle cells by hematoxylin and eosin (H&E) staining (Figure 2A), which aided in diagnosing the patient with spindle cell carcinoma of the lung. While gene mutation test results for epidermal growth factor receptor mutations and anaplastic lymphoma kinase rearrangement were negative, programmed death ligand-1 (PD-L1) expression was observed in more than 90% of the tumor cells of the lung biopsy tissue (Figure 2B) by immunohistochemistry (IHC) using the PD-L1 IHC 22C3 pharmDx antibody (clone 22C3; Dako North America Inc.). A positron emission tomography (PET)-CT scan showed fluorodeoxyglucose uptake in the mediastinal and hilar lymph nodes, right adrenal gland, and right trapezius muscle, which was consistent with his symptom (Figure 3A). Based on all these findings, the patient was diagnosed with advanced lung cancer, spindle cell carcinoma, cT4N2M1c, stage IVB.

From April 2017, the patient began receiving 200 mg pembrolizumab intravenously every 3 weeks. The lung and metastatic tumors dramatically decreased in size after 9 weeks, and tumor regression was maintained after 21 weeks, as indicated by the reduced fluorodeoxyglucose uptake observed in the PET-CT scan (Figure 3B). The tumor continued to reduce in size, and the patient’s complaint of neck pain was resolved.

## Discussion

Recently, patients with advanced NSCLC have demonstrated significantly longer overall survival after treatment with immune checkpoint inhibitors than those after standard chemotherapy. PD-L1 is a promising biomarker for immunotherapy. Multiple clinical trials suggest that PD-L1 expression in tumors predicts response to immune checkpoint inhibitors^(4), (5), (6), (7)^.

High PD-L1 expression in SC has been previously reported^(8)^, where subjects with SC were predominantly men (10 of 13) with an average age of 60.2 years. Of the 13 SC patients (including 1 spindle cell carcinoma), 9 (69.2%) were positive for PD-L1 expression, indicating a potential use for immune checkpoint inhibitors in SC.

While there have been some case reports on the dramatic and long-lasting efficacy of immune checkpoint inhibitors in pulmonary pleomorphic carcinoma, the most prevalent type of SC^(9), (10)^, there are no previous reports describing the use of pembrolizumab for spindle cell carcinoma of the lung. In this case, the limited specimen obtained from the transbronchial lung biopsy made the diagnosis challenging, and we relied on our pathologist’s judgment who diagnosed the patient with spindle cell carcinoma. While we acknowledge this diagnostic ambivalence, and the need for further investigation, our clinical results suggest that immunotherapy could be a promising treatment option for rare forms of lung cancer, including spindle cell carcinoma of the lung.

## Article Information

### Conflicts of Interest

Shunichi Sugawara received honorariums from AstraZeneca, Chugai Pharma, Pfizer, Taiho Pharmaceutical, Eli Lilly and Company, Novartis, Kyowa Hakko Kirin, Bristol-Myers Squibb, Ono Pharmaceutical, MSD K.K, Nippon Boehringer Ingelheim; Atsushi Nakamura received honorarium from MSD K.K; Yukihiro Toi received honorarium from MSD K.K.

### Acknowledgement

We thank Editage for their English editorial assistance.

### Author Contributions

Kyoji Tsurumi, Yosuke Kawashima, Ryohei Saito and Shunichi Sugawara managed the patient. Junichi Akahira evaluated the pathology slides. Yukihiro Toi, Atsushi Nakamura, Shinsuke Yamanda, Yuichiro Kimura and Yoshihiro Honda assisted preparation of the manuscript. All authors contributed to writing the report. Written consent for publication was obtained.

### Approval by Institutional Review Board (IRB)

This manuscript is a case report and does not need approval by IRB.

### Consent

Written informed consent was obtained from the patient publishing this case report and accompanying images.
